# Multiloculated Prostatic Abscess Complicated by Obstructive Hydronephrosis and Epididymo-Orchitis in an Undiagnosed Diabetic Patient: A Case Report

**DOI:** 10.7759/cureus.100256

**Published:** 2025-12-28

**Authors:** Colton Brewer, Eric H Chou

**Affiliations:** 1 Emergency Medicine, Baylor Scott & White Medical Center, Fort Worth, USA

**Keywords:** bacterial prostatitis, methicillin-sensitive staphylococcus aureus, obstructive hydronephrosis, prostatic micro abscess, purulent epididymo-orchitis, transurethral resection of prostate (turp)

## Abstract

Prostatic abscesses are rare but potentially fatal infections, particularly in immunocompromised patients such as those with uncontrolled diabetes. Complications like obstructive hydronephrosis and epididymo-orchitis are uncommon and may increase morbidity. We report the case of a 48-year-old man with no significant medical history who presented with several months of pelvic and scrotal pain. He was found to have new-onset diabetes mellitus (HbA1c >14%), and imaging revealed a 6 cm multiloculated prostatic abscess with right-sided hydroureteronephrosis and concurrent epididymo-orchitis. Initial CT-guided aspiration grew methicillin-sensitive *Staphylococcus aureus* (MSSA). Due to persistent infection and symptoms, the patient underwent transurethral resection and drainage. His course was further complicated by a catheter-associated urinary tract infection (CAUTI) caused by extended-spectrum beta-lactamase (ESBL) producing *Klebsiella pneumoniae*, which was successfully treated with intravenous ertapenem. This case underscores the importance of prompt recognition and aggressive management of prostatic abscesses, especially in patients with undiagnosed diabetes. Imaging, surgical drainage, and targeted antibiotic therapy are crucial in preventing complications and improving outcomes.

## Introduction

Prostatic abscess is an uncommon complication of acute bacterial prostatitis and remains a rare urologic diagnosis overall [1-3]. It typically arises in individuals with predisposing factors such as diabetes mellitus, immunosuppression, or recent urologic instrumentation [3,4]. While *Enterobacteriaceae*, particularly *Escherichia coli*, remain the predominant pathogens, more recent reports describe increasing involvement of methicillin-sensitive and methicillin-resistant *Staphylococcus aureus* (MSSA and MRSA), especially in patients with bloodstream infection and hematogenous seeding of the prostate (i.e., bacterial spread via the circulation), as well as polymicrobial infections [5-8].

Commonly reported complications of prostatic abscess include epididymo-orchitis, abscess rupture, and systemic sepsis. In contrast, extension causing ureteral compression with obstructive hydronephrosis is exceptionally rare and has only been described in isolated case reports, making it far less common than these other sequelae [9,10]. When present, hydronephrosis represents a serious consequence, with potential for upper urinary tract obstruction, acute kidney injury, and heightened risk of urosepsis, and may not be adequately managed with antibiotics and standard drainage alone.

Against this background, we present a rare case of a multiloculated MSSA prostatic abscess in an undiagnosed diabetic patient, complicated by unilateral hydronephrosis and epididymo-orchitis. This constellation of findings underscores the rarity of hydronephrosis as a complication, highlights the diagnostic challenges of prostatic abscess, and emphasizes the need for timely, aggressive, multidisciplinary management.

## Case presentation

A 48-year-old man with no known prior medical history presented to the emergency department with two months of right lower quadrant and right scrotal pain, associated with intermittent subjective fevers and chills. He had previously been evaluated in Mexico, where imaging reportedly raised concern for malignancy; however, he delayed follow-up due to a lack of insurance. He denied dysuria, hematuria, voiding difficulties, recent sexual activity, intravenous drug use, and any history of sexually transmitted infections.

On presentation, his vital signs were notable for a heart rate of 109 beats per minute and blood pressure of 147/99 mmHg. He was afebrile. Examination revealed right scrotal swelling and tenderness without overlying erythema, induration, or fluctuance. Abdominal, cardiopulmonary, and digital rectal examinations were otherwise unremarkable.

Laboratory testing demonstrated marked hyperglycemia (glucose 572 mg/dL; reference: 70-99 mg/dL), leukocytosis (white blood cell count 14.2×10³/µL; reference: 4.5-11.0×10³/µL), and elevated hemoglobin A1c (>14%; reference: <6.5%), consistent with previously undiagnosed diabetes mellitus. Renal function on initial labs was unremarkable with normal serum creatinine (1.18; reference: 0.70-1.30) and normal blood urea nitrogen, BUN (15; reference: 7-18). There was no anion gap (7; reference: >12) or metabolic acidosis with normal bicarbonate (26; reference: 22-28). Urinalysis showed 2+ leukocyte esterase (reference: negative) and 10-20 white blood cells/hpf (reference: 0-5). 

**Figure 1:**
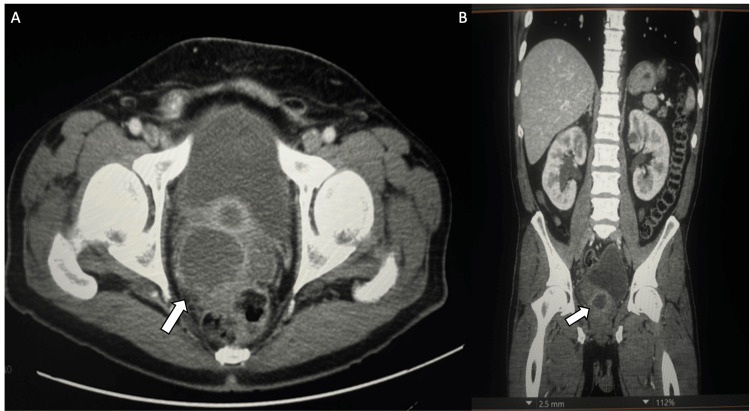
CT abdomen/pelvis with contrast revealed a 6 cm multiloculated prostatic abscess (arrow) A: axial view; B: coronal view

**Figure 2:**
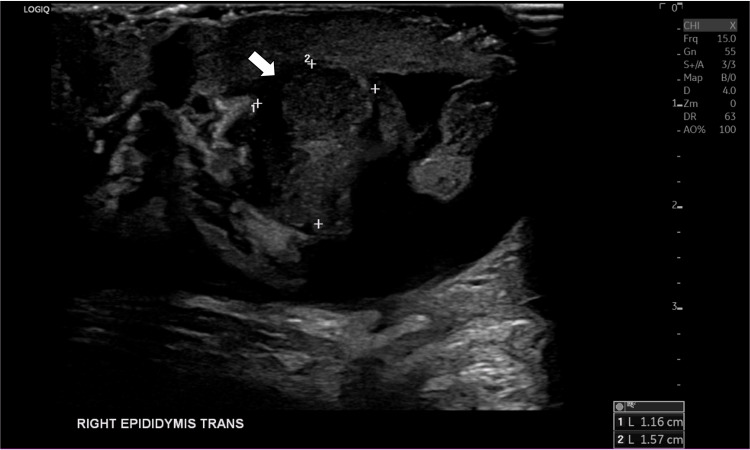
Scrotal ultrasound showed a concurrent right scrotal fluid collection and right epididymo-orchitis (arrow)

Contrast-enhanced CT of the abdomen and pelvis revealed a 6 cm multiloculated prostatic abscess extending into the right seminal vesicle with mass effect on the right ureter, resulting in right-sided hydroureteronephrosis (Figure 1). Scrotal ultrasound demonstrated a right-sided fluid collection with findings consistent with epididymo-orchitis (Figure 2).

Empiric intravenous piperacillin-tazobactam and vancomycin were initiated. CT-guided drainage of the prostatic abscess yielded only 2 mL of purulent material, as the abscess was multiloculated and difficult to aspirate. This 2 mL of purulent material cultured methicillin-sensitive *Staphylococcus aureus* (MSSA). Despite this intervention, the patient remained febrile with persistent leukocytosis and had postoperative hypotension. With stabilization of vitals, the patient subsequently underwent transurethral resection and drainage of the abscess the next day, during which approximately 100 mL of purulent fluid was evacuated. This purulent fluid again cultured MSSA. Pathology revealed benign prostatic hyperplasia with acute inflammation and no evidence of malignancy. A transthoracic echocardiogram showed no evidence of infective endocarditis. He was discharged with a peripherally inserted central catheter to complete outpatient antibiotic therapy with daptomycin 500 mg once daily and trimethoprim-sulfamethoxazole (Bactrim®) 800 mg/160 mg twice daily, completing a total 28-day course of antibiotics.

In the postoperative period, 15 days after surgery, he developed a catheter-associated urinary tract infection due to extended-spectrum β-lactamase (ESBL)-producing *Klebsiella pneumoniae*, which was treated with intravenous ertapenem. Follow-up imaging confirmed resolution of the prostatic abscess and right-sided hydroureteronephrosis.

## Discussion

This case highlights key aspects of the epidemiology, risk factors, microbiology, imaging, and management of prostatic abscess, while underscoring several unusual features: *Staphylococcus aureus *as the causative organism, associated hydroureteronephrosis, and a complicated postoperative course in the setting of poorly controlled diabetes.

As a percentage of all urologic conditions, prostatic abscess is exceedingly rare. There are no robust, recent epidemiologic studies quantifying its incidence among all urologic diagnoses, but expert reviews consistently describe it as a rare diagnosis in the broader urologic population. Approximately 2.7% to 6% of men with acute bacterial prostatitis will develop prostatic abscess [11-13]. The clinical presentation is often nonspecific and overlaps with acute prostatitis or urinary tract infection, which can delay diagnosis. Perineal or pelvic discomfort in combination with systemic signs of infection, such as fever and myalgias, should raise clinical suspicion, particularly when symptoms do not respond as expected to standard therapy [1,2]. In our patient, perineal pain and sepsis criteria in the context of uncontrolled diabetes were important early diagnostic clues.

Diabetes mellitus is the most consistently reported risk factor, present in over half of patients with prostatic abscess, and up to one quarter are diagnosed with diabetes during the workup [3,4]. Immunocompromised and poorly controlled diabetic patients, like ours, are more likely to demonstrate severe or atypical presentations and to develop systemic complications such as sepsis [5,7]. The hydroureteronephrosis observed in this case illustrates a particularly important, but rare, complication: urinary tract obstruction due to mass effect from an enlarged, inflamed prostate or abscess cavity. If unrecognized, this can progress to obstructive nephropathy or urosepsis [9].

The microbiology of prostatic abscess is evolving. Gram-negative bacilli, especially *Escherichia coli*, remain the most common pathogens, but *Staphylococcus aureus* is increasingly recognized, particularly in patients with hematogenous spread or immunocompromising conditions [5-7]. Gram-positive organisms such as MRSA and MSSA are often linked to distant foci of infection, including skin, soft tissue, or bone, with subsequent hematogenous seeding [6,7]. MSSA was isolated from our patient's abscess fluid, an uncommon but reported etiology in diabetic patients [8,9]. Historical sexually transmitted pathogens such as *Neisseria gonorrhoeae *and *Chlamydia trachomatis* have become less frequent in the antibiotic era [4], consistent with our patient's lack of sexual activity and absence of urethral symptoms.

Imaging is central to diagnosis and management. Both computed tomography (CT) and transrectal ultrasound (TRUS) can identify focal collections, define abscess size and loculation, and assess for complications [10,14]. Early imaging is crucial, as management typically requires prolonged antibiotic therapy, usually two to four weeks, and frequently procedural intervention [1-3]. Initial treatment generally consists of admission and broad-spectrum intravenous antibiotics tailored to culture results. Lesions larger than 2 cm, multiloculated collections, or abscesses that fail to respond to antibiotics alone usually warrant drainage. Image-guided aspiration via CT or TRUS is a preferred first-line approach; however, transurethral resection or unroofing may be required in complex or refractory cases [15,16]. In our patient, a persistent abscess on CT despite aspiration necessitated TURP, illustrating the need for timely escalation when minimally invasive measures are unsuccessful.

Beyond local mass effect and hydronephrosis, prostatic abscess can extend into adjacent structures such as the seminal vesicles, perineum, or rectum, leading to fistula formation or rupture into these spaces [9,17]. Epididymo-orchitis, although rare, has also been reported as a complication due to contiguous spread along the genitourinary tract [18]. Systemic complications-bacteremia, sepsis, septic shock, and multi-organ failure-are more frequent in patients with diabetes or other immunocompromising conditions [7]. Our patient's postoperative course further underscores the vulnerability of this population: he developed a catheter-associated urinary tract infection due to ESBL-producing *Klebsiella pneumoniae*, which required carbapenem therapy. This highlights the importance of meticulous catheter care, rigorous infection control practices, and antimicrobial stewardship when managing these patients [19,20].

Clinically, this case reinforces the need for a high index of suspicion for prostatic abscess in patients with pelvic or perineal pain, systemic infection, and relevant risk factors such as uncontrolled diabetes, particularly when presumed prostatitis or urinary tract infection does not improve as expected. It also illustrates the importance of recognizing evolving microbiology, including *S. aureus* (MSSA and MRSA), tailoring antibiotic therapy to culture results, using early imaging and prompt urologic involvement to detect complications such as obstruction or hydroureteronephrosis, and anticipating both abscess-related complications and nosocomial infections associated with invasive devices and prolonged hospitalization.

## Conclusions

Prostatic abscess should be suspected in patients with pelvic or perineal pain and systemic signs of infection, particularly in those with undiagnosed or poorly controlled diabetes. Early imaging and microbiologic diagnosis are essential to prevent diagnostic delays that may allow progression to rare but serious complications such as hydronephrosis and epididymo-orchitis, with potential consequences for renal function and fertility. This case highlights the importance of timely surgical intervention when medical therapy is insufficient, along with close monitoring for evolving obstructive or infectious sequelae. It also underscores the value of multidisciplinary care, including urology, radiology, and infectious diseases, and of antimicrobial stewardship to ensure appropriate, culture-directed therapy that balances effective source control with minimizing unnecessary broad-spectrum antibiotic exposure.
